# *Pneumocystis* Colonization Is Associated with Enhanced Pulmonary Remodeling and Activation of Redox-Responsive Pathways in a COPD Experimental Model

**DOI:** 10.3390/antiox15050526

**Published:** 2026-04-22

**Authors:** Andrea Méndez, Krishna Coronado, Diego A. Rojas

**Affiliations:** 1 Escuela de Kinesiología, Facultad de Salud y CIencias Sociales, Campus Providencia, Sede Santiago, Universidad de Las Américas, Santiago 7500975, Chile; mmendezg@udla.cl; 2 Centro de Investigación en Ciencias Biológicas y Químicas, Universidad de Las Américas, Santiago 7500975, Chile; 3 Instituto de Ciencias Biomédicas (ICB), Facultad de Ciencias de la Salud, Universidad Autónoma de Chile, Santiago 8910132, Chile; krishna.coronado@cloud.uautonoma.cl

**Keywords:** *Pneumocystis*, mucus, COPD, inflammation, antioxidants, oxidative stress, mucins, fibrosis, Nrf2

## Abstract

Chronic Obstructive Pulmonary Disease (COPD) is characterized by persistent inflammation and structural alterations in the lung triggered mainly by oxidative stress. Colonization by the opportunistic fungus *Pneumocystis* has been associated with worse clinical outcomes in COPD, yet its role in airway remodeling remains unclear. To this end, an elastase-induced COPD model was established, followed by colonization with *Pneumocystis*. Lung tissue was analyzed histologically and molecularly to assess epithelial thickness, alveolar morphometric parameters (mean linear intercept [MLI], D_0_, D_1_, D_2_), inflammation, collagen deposition, and the expression of remodeling and oxidative stress markers. Emphysematous damage parameters MLI, D_0_, D_1_, and D_2_ were markedly elevated in co-exposed animals, indicating enhanced alveolar enlargement. Animals with COPD and *Pneumocystis* colonization showed a significant increase in airway inflammation compared with control, COPD, and *Pneumocystis* groups. Airway epithelial thickness, mucus metaplasia, and collagen deposition exhibited a summative increase in the COPD/*Pneumocystis* group. Redox-responsive markers, such as superoxide dismutase (SOD) and catalase, were upregulated. Moreover, protein and mRNA levels of nuclear factor erythroid 2–related factor 2 (Nrf2) and its downstream gene heme oxygenase-1 (Hmox1) were significantly increased, with the strongest activation observed in co-exposed animals. Integrative correlation analysis showed that *Pneumocystis* burden positively correlated with lung damage, inflammation, and epithelial remodeling. These structural alterations were accompanied by coordinated activation of the antioxidant pathway Nrf2. Taken together, *Pneumocystis* colonization is associated with enhanced pulmonary remodeling and modulation of antioxidant signaling in experimental COPD, promoting structural and molecular changes that may contribute to disease progression. These findings suggest that *Pneumocystis* acts as an amplifying factor in COPD-associated lung damage.

## 1. Introduction

Chronic Obstructive Pulmonary Disease (COPD) is characterized by chronic inflammation, airway remodeling, and progressive parenchymal destruction, leading to marked airway obstruction [1,2,3]. COPD ranks among the major contributors to global morbidity and mortality, with an increasing incidence associated with population aging, smoking, and urban pollution [4,5,6]. Additionally, airway epithelial remodeling is a key process underlying the clinical manifestations of COPD [7,8,9]. Remodeling occurs when the airway epithelium undergoes profound architectural and functional alterations in response to cigarette smoke, environmental pollutants, and microbial-derived signals. These changes are characterized by epithelial thickening, goblet cell hyperplasia, and mucus metaplasia [10,11,12]. In this context, chronic inflammation is a key process that, through the release of cytokines and proteases by macrophages and neutrophils, directly or indirectly affects airway remodeling, increasing profibrotic pathways such as TGFβ1 or goblet cell hyperplasia [13,14,15].

The main driver of the epithelial damage in COPD is oxidative stress [16]. The lungs of individuals with COPD have been exposed to sustained oxidative challenges from exogenous sources, such as cigarette smoke, and from endogenous sources, including activated macrophages [17] and neutrophils [18]. Excessive reactive oxygen species (ROS) contribute to cellular apoptosis [19], lipid peroxidation [20], mucus hypersecretion [21,22], overexpression of inflammatory markers [23], and disruption of the extracellular matrix homeostasis [24]. The host’s antioxidant defenses, comprising enzymes such as catalase, superoxide dismutase (SOD), and Hmox1, as well as transcriptional regulators such as Nrf2 and FOXO3, attempt to counteract oxidative insults [19,25]. In COPD, these pathways are affected due to modifications or changes in the gene expression of the oxidative stress-related transcription factors Nrf2 and FOXO3, leading to the epithelial damage of the airways [26,27].

The progression of the disease is deeply influenced by chronic airway infections, exacerbating the disease’s symptoms [28,29,30]. Among the nontraditional microbial agents implicated in chronic airway diseases, *Pneumocystis* has emerged as a relevant organism. *Pneumocystis* is a fungal pathogen associated with pneumonia [31] with strict host specificity and a unique biology characterized by airborne transmission and a predilection for the alveolar spaces [32,33]. Its ability to colonize the lung without necessarily causing pneumonia has been described in recent years [34,35,36]. Importantly, several clinical studies have documented an elevated prevalence of *Pneumocystis* colonization in patients with COPD [37,38,39], and this prevalence correlated with the disease severity [40]. *Pneumocystis* cell wall components activate the innate immune response in the airways, leading to NF-κB-mediated inflammation [41,42,43]. In addition, interaction of *Pneumocystis* with the host stimulates several other immune responses, including Th1 [44,45], Th2 [46,47,48,49], and Th17 [45,50,51], establishing a robust host immune response and activating distinct inflammatory pathways and increasing mucin expression on airway surfaces, as observed in humans [34] and animal models [48,49,52]. Moreover, *Pneumocystis* colonization induces a mild airway remodeling phenotype, as documented in primary infection rat models characterized by mucus metaplasia, increased airway epithelial thickness, and a slight increment of peribronchial collagen deposition [48,49].

Animal models that mimic COPD have been widely used [53,54]. One of them is elastase-induced COPD, which replicates key histopathological features of the disease, including alveolar enlargement, destruction of distal airspaces, and progressive collagen deposition [55,56,57]. Interestingly, when this COPD animal model is combined with *Pneumocystis* colonization, the classical features of the disease are increased synergistically as documented [58,59]. This evidence supports the hypothesis that mild *Pneumocystis* infections may exacerbate the damage caused by COPD progression. However, there is no evidence linking *Pneumocystis* colonization and airway remodeling. In addition, respiratory infections have been shown to induce oxidative stress, leading to a modulation of antioxidant pathways such as Nrf2 [60], but the mechanisms underlying this response in *Pneumocystis* have not been described.

Therefore, the goal of this study was to evaluate the combined impact of elastase-induced COPD and *Pneumocystis* colonization in a rat model, focusing on summative effects on airway airspace enlargement, inflammation, airway thickness, and mucus metaplasia. In addition, we examined the modulation of the Nrf2 pathway and downstream antioxidant-associated genes. To this end, we performed histological and molecular analyses to evaluate structural remodeling, mucus production, and markers of redox-response signaling.

## 2. Materials and Methods

### 2.1. Ethics

Animal experiments were performed at the Faculty of Medicine of the University of Chile, according to protocol CBA#1110, approved by the Institutional Committee for Care and Use of Animals (CICUA), under certification N° 19336-MED-UCH. Experiments in animals were conducted in agreement with the Animal Protection Law 20,380 of Chile and the Guide for the Care and Use of Laboratory Animals (8th Edition, National Academies Press, Washington, DC, USA). Animals were housed under standard conditions, and animal welfare was evaluated using the Morton and Griffiths test.

### 2.2. Elastase-Induced COPD Animal Model and Pneumocystis Infection

**Figure 1:**
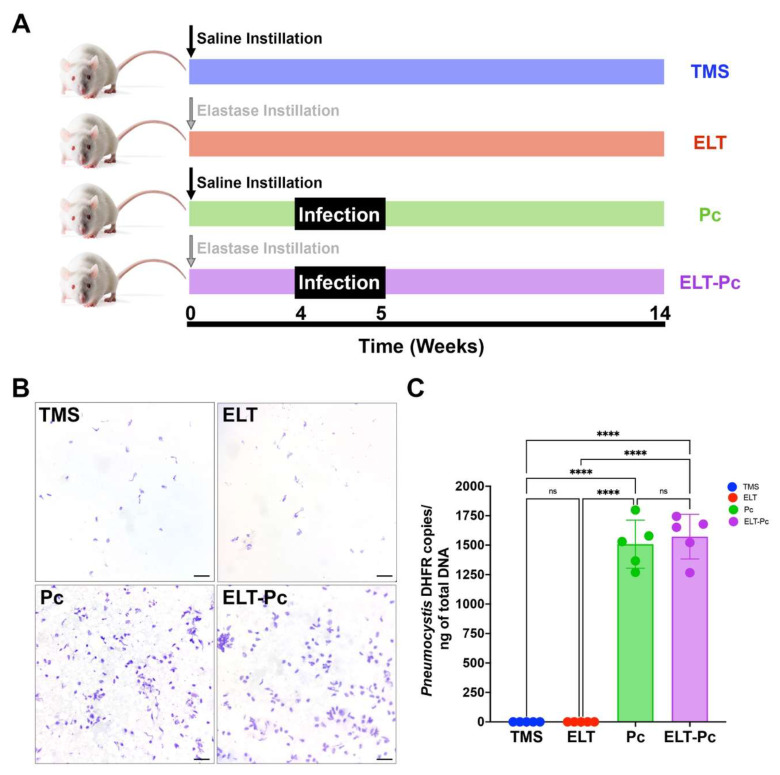
Detection of *Pneumocystis* colonization in the elastase COPD-induced animal model. (**A**) Scheme of the experimental model, including the four animal groups: the control group treated with trimethoprim/sulfamethoxazole to prevent *Pneumocystis* colonization (TMS), the COPD-only group (ELT) treated with elastase to induce COPD, the *Pneumocystis* group (Pc) infected with *Pneumocystis*-pneumonia (PcP) animals and the COPD/*Pneumocystis* group (ELT-Pc) treated with elastase and infected with PcP rats. Infections were induced 4 weeks after instillations for one week. The experiment took 14 weeks in total. (**B**) Representative images showing *Pneumocystis* cysts detected in lung impressions smears stained with cresyl violet. The bar scale represents 20 μm. (**C**) *Pneumocystis* burden was determined by qPCR detection of the single-copy *dhfr* gene and normalized to total DNA in each sample. The detection limit was 1 *Pneumocystis* DHFR copy/ng. Data were expressed as mean ± SD and analyzed by ANOVA. ns = non-significant; **** = *p* < 0.0001.

Animal models presented in this work are a replication of the previously published model [59]. Briefly, Sprague–Dawley female rats weighing 300 g, were assigned randomly to groups of 3 animals per cage using http://www.randomizer.org. COPD was induced in half of the animals by the instillation of 1 U of elastase (SIGMA, St. Louis, MO, USA). Control animals received only a saline buffer. To prevent *Mycoplasma* infections, Tylosine (1 g/L) was added to the drinking water. This treatment was applied for 5 weeks, starting 1 week before elastase instillation. *Pneumocystis* infection was induced 4 weeks after instillation using the co-housing approach [49,58,59]. To this end, *Pneumocystis carinii*-infected animals were contacted with non-colonized animals in a 1:3 proportion. These animals were put in the cages for 1 week. Non-colonized animals were treated with trimethoprim–sulfamethoxazole (TMS) (TMP-SMZ; 80 mg TMP and 400 mg SMZ per 5 mL) 15 mL/L in the drinking water to prevent the growth of the fungus. In total, four experimental groups were generated according to elastase instillation and colonization, and their respective controls: control animals without elastase instillation and non-colonized (TMS); COPD elastase-induced animals and non-colonized (ELT); and COPD elastase-induced animals and colonized with *Pneumocystis* (ELT-Pc). Each animal group consisted of 5–6 animals. A summary of the animal model is presented in Figure 1A. All the experiments were stopped 14 weeks after instillation. The animals’ lungs were perfused with cold PBS before extraction. The left lobule was inflated by instillation with 3.7% formalin buffered with PBS (pH 7,4) at a constant pressure of 25 cm water above each animal for 10 min. Then, the left lobules were fixed in formalin-filled vessels for 48 h at 4 °C. Right lobules were quickly frozen at −80 °C.

### 2.3. Detection of Pneumocystis

Evaluation of *Pneumocystis* burden was performed by measuring the copy number of the *dhfr* gene. To this end, quantitative PCR was used. Genomic DNA was extracted from 20 mg of lung tissue using the Genomic DNA Isolation Kit (Norgen, Thorold, ON, Canada). 200 ng of genomic DNA were used to amplify the *dhfr* gene with the following protocol: an initial denaturation step of 15 min at 95 °C, followed by 40 cycles of 15 s at 95 °C, 15 s at 55 °C, and 15 s at 72 °C. These experiments were performed using Brilliant II SYBR Green QPCR Master Mix (Agilent, Santa Clara, CA, USA) and the AriaMx qPCR instrument (Agilent, Santa Clara, CA, USA). To determine the *Pneumocystis* burden, amplification of the same *dhfr* section previously inserted in a pGEM-T Easy plasmid (Promega, Madison, WI, USA) was used as a reference. The detection limit was estimated at 1 copy/ng. The primers used to amplify the *dhfr* gene are shown in Table 1. *Pneumocystis* cysts were detected by cresyl violet staining (ab246817, Abcam, Cambridge, UK) in lung impression smears prepared by direct contact of lung tissues from each animal. Smears were fixed at 70 °C, and a drop of cresyl violet was added to the slide. After 5 min of incubation, the slides were washed with distilled water and air-dried. Mounted slides were observed under an Olympus BX60 optical microscope (Olympus, Tokyo, Japan).

### 2.4. Histology

Fixed lungs were processed using an alcohol/xylol battery using the following protocol: 30 min in alcohol 90%, 30 min in alcohol 96%, 30 min in alcohol 100% I, 30 min in alcohol 100% II, 30 min in alcohol 100% III, 30 min in xylol I, 30 min in xylol II, 1 h in xylol III. Then, processed lungs were impregnated in paraffin at 65 °C according to the following protocol: 1 h in paraffin I, 15 h in paraffin II, and 1 h in paraffin III. After this point, the paraffin blocks were made. 5 μm-thick sections were obtained, processed, and stained with either hematoxylin and eosin (H/E), Alcian blue (AB), or picrosirius red, following the described procedures by the manufacturers (DIAPATH, Martinengo, Italy). Images were captured using an Ocus^®^20 microscope slide scanner (Grundium, Tampere, Finland) and opened in the free software Qupath version 0.6.0 [61]. Image processing was performed in ImageJ software v.1.54 (NCBI, Bethesda, MD, USA). To assess space enlargement in the samples, the mean linear intercept (MLI) and the D_0_, D_1_, and D_2_ parameters were measured in H/E-stained images. First, to determine MLI, five calibrated images (285 µm height × 252 mm width) from each animal in each experimental group were opened in ImageJ. A grid of 9 horizontal lanes and 10 vertical lanes was added to each calibrated image, and the intercepts were measured. The length of the grid was divided by the total number of intercepts of alveolar walls with the lanes according to the following formula: MLI = [(height × 9) × (width × 10)]/N° of intercepts. D_0_, D_1_, and D_2_ parameters were determined according to previously described [62,63]. To this end, the equivalent airspace diameter was measured. Briefly, the same images used to determine MLI were opened in ImageJ, calibrated, converted to 8-bit, and thresholded to produce a binary black/white image. Background and noise were reduced in each image. An inverted mask was made with the objects of interest in black. Airspaces were delimited using the watershed transform. The airspace area (A_i_) was measured in each alveolus, counted as a particle in the software. Then, the equivalent airspace diameter was measured by the formula d_i_ = 2 × √(A_i_/π). D_0_ was determined as the arithmetic mean of all d_i_ values for each image. The variance and skewness were determined for each animal from the entire set of images evaluated. D_1_ and D_2_ parameters were generated as previously described [62]. All these morphometric parameters are expressed in μm. Peribronchiolar inflammation was assessed as previously described [58] by quantifying the percentage of inflamed airways per animal. Airway epithelial thickness was determined by measuring the area between the lumen and the basal membrane in H/E-stained images opened in ImageJ. Each area was normalized to the airway perimeter determined by measuring the length of the basal membrane. The results of these parameters were expressed in μm. The AB-stained sections were used to evaluate two parameters: AB-positive area and the mean linear density (MLD) of AB-positive cells. To this end, the bronchiolar epithelium occupied by mucus was measured, determining the percentage of the area delimited by lumen and the basal membrane, and considering only the AB-positive region. The AB-positive cell density was determined by counting positive cells in the AB-positive area and normalizing the count to the perimeter (cells/mm). 120 µm^2^ was used as the standard single-cell area. Peribronchial collagen was determined by picrosirius staining (DIAPATH, Martinengo, Italy) according to the manufacturer’s protocol. The positive area was normalized to the perimeter of each airway and expressed in μm. At least seven bronchioles per animal were analyzed in all the experiments.

### 2.5. Gene Expression Determinations

The expression of inflammation and redox-response genes was evaluated by qPCR. To this end, total RNA was obtained from 20 mg of frozen lung tissue, which was homogenized with 500 μL of RNA solv (Omega Biotek, Norcross, GA, USA). Then, 100 mL of chloroform was added to each tube and mixed by vortex. The aqueous phase was obtained by cold centrifugation at 14,000× *g* for 15 min. Precipitation of RNA was made by adding 250 μL cold isopropyl alcohol to the aqueous phase in a new tube. Mixes were frozen at −20 °C for an hour. RNA collection was performed by cold centrifugation at 14,000× *g*. Then, precipitated RNA was washed with 70% ethanol. Finally, after another centrifugation, the RNA was dried and suspended in 50 µL of nuclease-free water. DNA contaminations were eliminated by incubating 2 μg of each RNA with DNase (Thermo Fisher Scientific, Waltham, MA, USA), following the manufacturer’s instructions. After this, RNAs were retrotranscribed using M-MLV reverse transcriptase to obtain cDNAs following the manufacturer’s instructions (Promega, Madison, WI, USA). Single-stranded cDNAs were diluted with 75 μL of nuclease-free water, and 2 μL were used in qPCR experiments. Redox-response genes Catalase, SOD, Foxo3, Hmox1, and Nrf2 were amplified using Brilliant II SYBR Green QPCR Master Mix (Agilent, Santa Clara, CA, USA) and the AriaMx qPCR instrument (Agilent, Santa Clara, CA, USA). The amplification protocol used was as follows: an initial hot start at 95 °C for 12 min, followed by 45 cycles of 20 s at 95 °C, 20 s at 58 °C, and 20 s at 72 °C. Actin amplification was used as an internal control. Results were expressed as fold changes, normalized by actin, and referenced to the control group using the 2^−ΔΔCt^ method [64]. Primer sequences used in this work are presented in Table 1.

### 2.6. Statistics

All statistical analyses and plots were made using GraphPad Prism 10 (GraphPad Software Inc., San Diego, CA, USA). Variances and skewness were determined using column statistics to estimate parameters D_1_ and D_2_. All the results were expressed as mean ± standard deviation (SD). The experimental groups consisted of 5 animals, and each point in the plot represents the mean of several measurements. Data distribution was evaluated using the Shapiro–Wilk test, and group differences were analyzed using a two-way ANOVA. Multiple comparison was evaluated using the Tukey test. A *p*-value less than 0.05 was defined as statistically significant. Correlation analyses were performed using group-level mean values and were intended as tools to visualize coordinated trends among variables with an exploratory aim. As such, no statistical inference or *p*-value–based interpretation was applied. A data table was generated in GraphPad Prism containing the mean values for each variable across all experimental groups. Pairwise correlations between variables were calculated using the Pearson correlation coefficient (r). The resulting correlation matrix was visualized as a heatmap, with correlation coefficients color-coded by magnitude and direction.

## 3. Results

### 3.1. Colonization of Pneumocystis Elastase-Induced COPD Animals

We previously characterized this COPD-induced elastase animal model colonized with *Pneumocystis* [58,59]. To validate this replicated model, some documented features were evaluated. *Pneumocystis* identification was evaluated by quantifying fungal burden. To this end, the single-copy gene dihydrofolate reductase (*dhfr*) was measured by qPCR. As shown in Figure 1B, *Pneumocystis* was identified only in the animal groups that were co-housed with previously infected animals (Pc and ELT-Pc). These results are consistent with the direct detection of *Pneumocystis* cysts in lung smears, where *Pneumocystis* cysts were identified only in the Pc and ELT-Pc groups (Figure 1C).

### 3.2. Increase in Emphysematous Lung Damage in Elastase-Induced COPD Animals Is Enhanced by Pneumocystis Colonization

**Figure 2:**
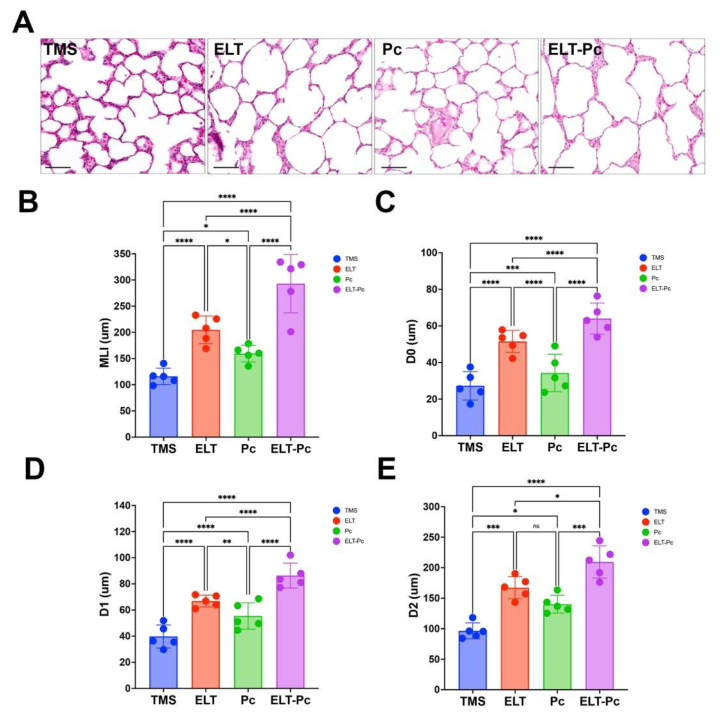
Evaluation of emphysematous injuries in elastase COPD-induced animals colonized with *Pneumocystis*. (**A**) Illustrative H/E-stained images of lung sections showing alveolar airspaces. The scale represents 50 μm. Emphysematous injuries were evaluated and quantified by applying the mean linear intercept (MLI) parameter (**B**) and equivalent airspace diameter-associated indices: D_0_ (**C**), D_1_ (**D**), and D_2_ (**E**). Data were expressed as mean ± SD and analyzed by ANOVA. ns = non-significant; * = *p* < 0.05; ** = *p* < 0.01; *** = *p* < 0.001; **** = *p* < 0.0001.

To determine the role of *Pneumocystis* colonization on COPD-associated lung injury, histological analysis and morphometric quantification were performed. Representative (H/E)-stained lung sections revealed a marked airspace enlargement in elastase-treated (ELT) animals compared with control rats (TMS), as shown in Figure 2A. In addition, *Pneumocystis*-colonized animals (Pc) showed a moderate structural alteration. Interestingly, animals exposed to *Pneumocystis* and elastase (ELT-Pc) exhibited the most severe disruption of alveolar architecture, characterized mainly by the pronounced airspace enlargement (Figure 2A). Quantitative analyses were then performed to confirm these observations. Mean linear intercept (MLI), a classical indicator of alveolar enlargement, was significantly increased in ELT animals compared with control TMS animals (Figure 2B).

In contrast, colonized animals Pc showed a moderate increase compared with controls (Figure 2B). Notably, the ELT-Pc animal group showed the highest MLI values compared with all the other experimental animal groups (Figure 2B), indicating an exacerbated emphysematous damage in the lungs of these animals associated with the combined effect of elastase and *Pneumocystis* colonization. In addition to MLI, emphysematous changes in the lungs of these animals were further characterized using D_0_, D_1_, and D_2_ parameters, which provide complementary information on the distribution and heterogeneity of airspace enlargement. Analysis of the D_0_ parameter showed a significant increase in airspace size in ELT animals compared with control animals, whereas colonized animals (Pc) showed only a modest increase (Figure 2C). The combined effect of *Pneumocystis* and elastase resulted in a pronounced increase in D_0_ compared with all other groups (Figure 2C), supporting a summative effect of elastase and *Pneumocystis* on airspace enlargement. The same results were observed when the D_1_ and D_2_ parameters were evaluated. These parameters are associated with the heterogeneity and severity of the emphysematous damage. Both parameters were elevated in ELT animals compared with controls (Figure 2D,E), whereas colonized animals showed a moderate increase, like D_0_. However, ELT-Pc animals showed a significantly greater increase in both D_1_ and D_2_ parameters, indicating a summative effect of COPD induction and *Pneumocystis* colonization on the heterogeneity and severity of emphysematous lung damage (Figure 2D,E). Taking all these results together, while elastase or *Pneumocystis* alone induces emphysematous changes in the lungs of these animals, the combined impact of both factors results in a marked exacerbation of the damage, supporting an interaction between COPD and fungal colonization in the induction of the emphysema.

### 3.3. Pneumocystis Colonization Increases the Peribronchial Inflammation in Elastase-Induced COPD Animals

**Figure 3:**
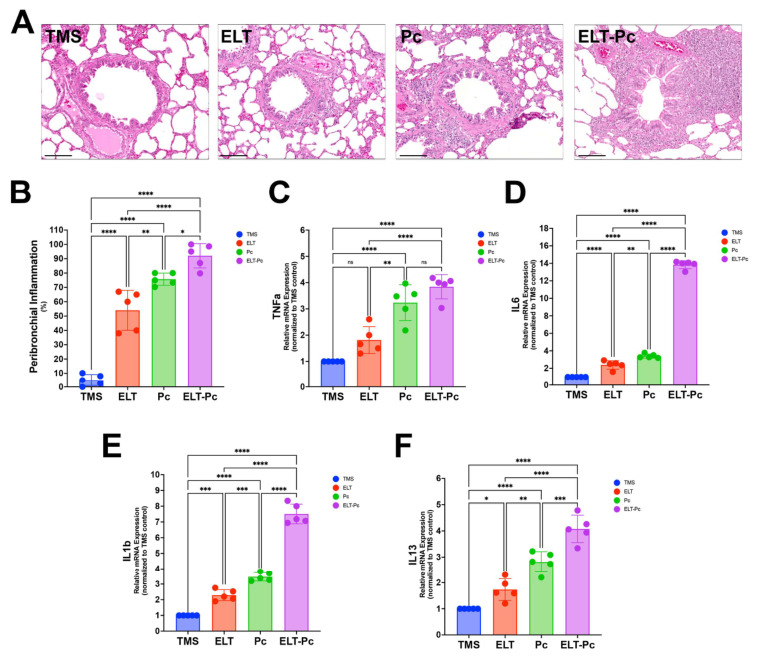
Detection of inflammation in elastase COPD-induced animals colonized with *Pneumocystis*. (**A**) Representative H/E-stained images of lung sections showing peribronchial inflammation. The bar scale represents 100 μm. (**B**) Peribronchial inflammation was quantified by measuring the percentage of airways in each animal that were inflamed. TNFα (**C**), IL6 (**D**), IL1β (**E**), and IL13 (**F**) mRNA levels were measured by qPCR. Relative mRNA expression of each gene was determined using the 2^−DDCt^ method. Data were expressed as mean ± SD and analyzed by ANOVA. ns = non-significant; * = *p* < 0.05; ** = *p* < 0.01; *** = *p* < 0.001; **** = *p* < 0.0001.

Previous studies in this COPD-induced elastase model documented increased airway inflammation and elevated levels of inflammatory markers in animals colonized by *Pneumocystis* and instilled with elastase. Since chronic inflammation is a key factor in airway remodeling, we evaluated peribronchial inflammation and some relevant markers in this new model. Histological analysis of H/E-stained lung sections revealed increased inflammatory infiltrates around the airways across experimental groups (Figure 3A). Compared with control animals (TMS), COPD-induced animals (ELT) and *Pneumocystis*-colonized animals (Pc) exhibited a significant increment in peribronchial inflammation relative to controls. Interestingly, animals co-exposed to elastase and *Pneumocystis* (ELT-Pc) showed the greatest increase in inflammation compared with the other groups (Figure 3B). In addition, expression of inflammation markers such as TNFα, IL6, and IL8 showed a summative increase in COPD animals exposed to *Pneumocystis*, recapitulating previous findings. Moreover, we previously documented the role of the Th2 immune response in *Pneumocystis* colonization [48,49], in which Th2 markers were upregulated. For this reason, we measured IL-13 expression in this new animal model, which showed increased IL-13 mRNA levels in all experimental groups compared with control animals. However, the COPD animals exposed to *Pneumocystis* showed a greater increase than all the animal groups (Figure 3F). These results corroborate the notion that *Pneumocystis* colonization may enhance airway inflammation, which is relevant to the observed changes in COPD and is associated with other features such as airway remodeling.

### 3.4. Pneumocystis Colonization Increases the Airway Epithelium Thickness in Elastase-Induced COPD Animals

**Figure 4:**
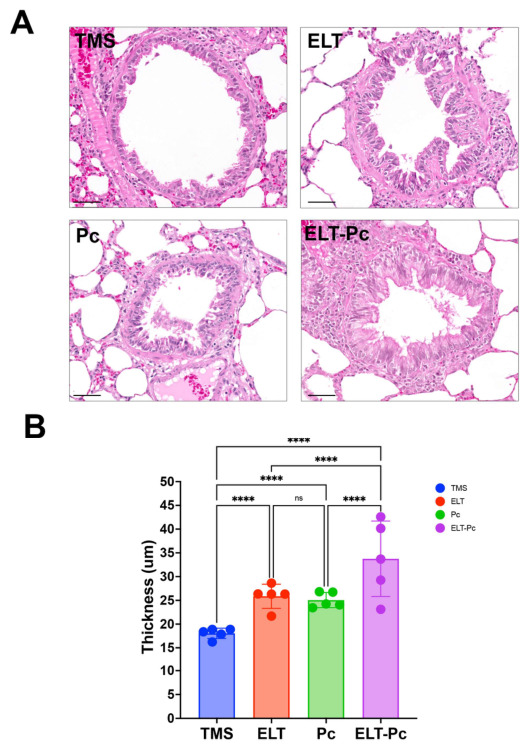
Detection of airway epithelium thickness in elastase COPD-induced animals colonized with *Pneumocystis*. (**A**) Representative H/E-stained images of lung sections showing airway epithelium thickness. The bar scale represents 50 μm. (**B**) Quantification of the airway epithelium thickness was determined by measuring the epithelial area between the basal membrane and the luminal surface of each analyzed airway. Data were expressed as mean ± SD and analyzed by ANOVA. ns = non-significant; **** = *p* < 0.0001.

To explore the role of *Pneumocystis* in airway remodeling, we first evaluate epithelial thickness in this model. Histological analysis of H/E-stained lung sections revealed alterations in airway epithelial architecture across experimental groups (Figure 4). Compared with control animals (TMS), COPD-induced animals (ELT) exhibited a significant increase in airway thickness, consistent with COPD-related epithelial remodeling. *Pneumocystis*-colonized animals (Pc) also displayed a moderate but significant increase in epithelial thickness relative to controls, whereas no significant difference was observed between ELT and Pc groups. Interestingly, animals exposed to both elastase and *Pneumocystis* (ELT-Pc) showed the most pronounced epithelial thickening with values significantly higher than those of all other groups (Figure 4). These results indicate that *Pneumocystis* colonization may enhance airway epithelial thickening, consistent with airway remodeling.

### 3.5. Pneumocystis Is Associated with Mucus Increment in the Airways of Elastase-Induced COPD Animals

**Figure 5:**
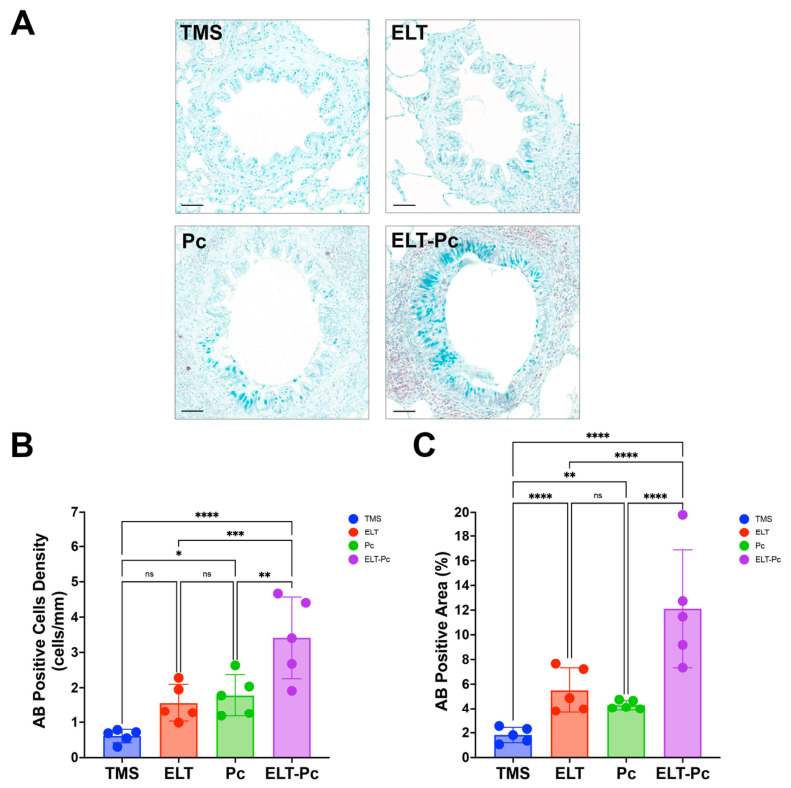
Evaluation of mucus metaplasia in the elastase COPD-induced *Pneumocystis* colonized animal model. (**A**) Representative images of AB-stained lung sections showing the presence of mucus in the epithelial airways. Scale bar represents 50 μm. Mucus metaplasia was quantified by determining the mean linear density (MLD) of AB-positive cells (**B**) and AB-positive epithelial area (**C**). Data were expressed as mean ± SD and analyzed by ANOVA. ns = non-significant; * = *p* < 0.05; ** = *p* < 0.01; *** = *p* < 0.001; **** = *p* < 0.0001.

Another feature related to airway remodeling is mucus metaplasia. To evaluate this parameter, we evaluated the presence of mucus in the airways. To this end, AB staining of lung sections revealed marked differences in mucus abundance among the experimental groups (Figure 5A). TMS animals showed a low density of AB-positive cells and a minimal stained area within the airway epithelium (Figure 5B,C). Animals exposed to elastase showed a moderate but significant increment in the AB-positive area compared with control animals. In contrast, Pc animals displayed a mild increase in mucus-producing cells and stained area compared with the TMS group, but with no differences compared with the ELT group (Figure 5B,C). Interestingly, animals exposed to elastase and *Pneumocystis* exhibit the most pronounced changes, showing a marked increase in the density of AB-positive cells and a significant increment of the AB-positive epithelial area in the airway epithelium compared with the other experimental groups (Figure 5B,C). The combined increase in AB-positive cell density and epithelial AB-stained area suggests a mucus metaplasia-like response, exacerbated when *Pneumocystis* is present in the airways of COPD animals, supporting evidence of airway remodeling and consistent with previously documented evidence [58,59].

### 3.6. Pneumocystis Is Associated with the Increment of Peribronchial Collagen Deposition in Elastase-Induced COPD Animals

**Figure 6:**
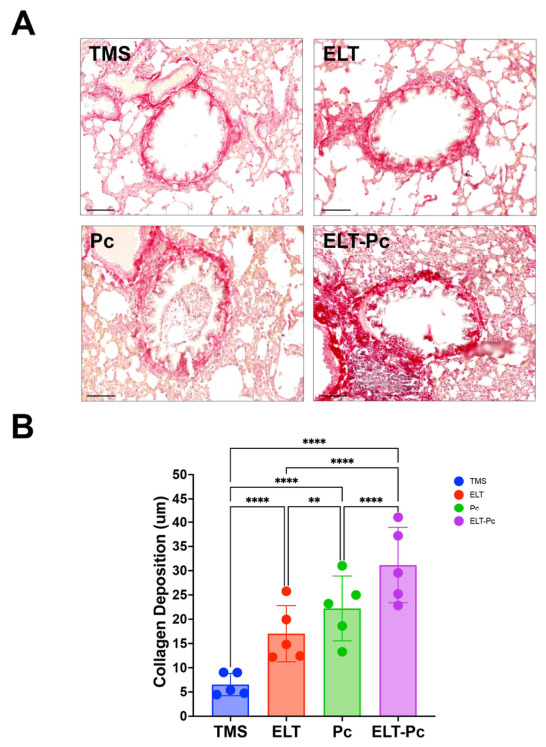
Identification of peribronchiolar collagen in elastase COPD-induced animals colonized with *Pneumocystis*. (**A**) Representative images of picrosirius red-stained lung sections showing collagen deposition around airways. Scale bar represents 50 μm. (**B**) Quantification of the occupied airway epithelial area by collagen. The picrosirius red-positive area was determined between the basal membrane and the luminal surface of each airway and normalized to the basal membrane perimeter. Data were expressed as mean ± SD and analyzed by ANOVA. ** = *p* < 0.01; **** = *p* < 0.0001.

Changes in the extracellular matrix are associated with airway remodeling. To this end, peribronchial collagen deposition was evaluated using picrosirius red-stained lung sections. As shown in Figure 6, the staining revealed marked differences between the experimental groups. Lung sections from TMS animals showed minimal collagen accumulation around the airways, consistent with standard airway architecture. In contrast, animals treated with elastase showed a significant increase in collagen deposition in the peribronchial region, consistent with airway remodeling associated with COPD-like damage. Colonized animals (Pc) also exhibited a substantial increase in peribronchial collagen deposition compared with controls, although with higher values compared with the ELT group. Notably, collagen deposition in the ELT-Pc group was significantly greater than that observed in either single-hit condition (ELT or Pc), suggesting that *Pneumocystis* colonization exacerbates the extracellular matrix remodeling around the airways. These results indicate an association between *Pneumocystis* and more severe COPD features, specifically a more severe airway remodeling phenotype. These results are consistent with those previously documented in this model and in the *Pneumocystis* primary infection model [49,58,59].

### 3.7. Increase in Redox-Responsive Markers Is Associated with Pneumocystis Colonization in Elastase-Induced COPD Animals

**Figure 7:**
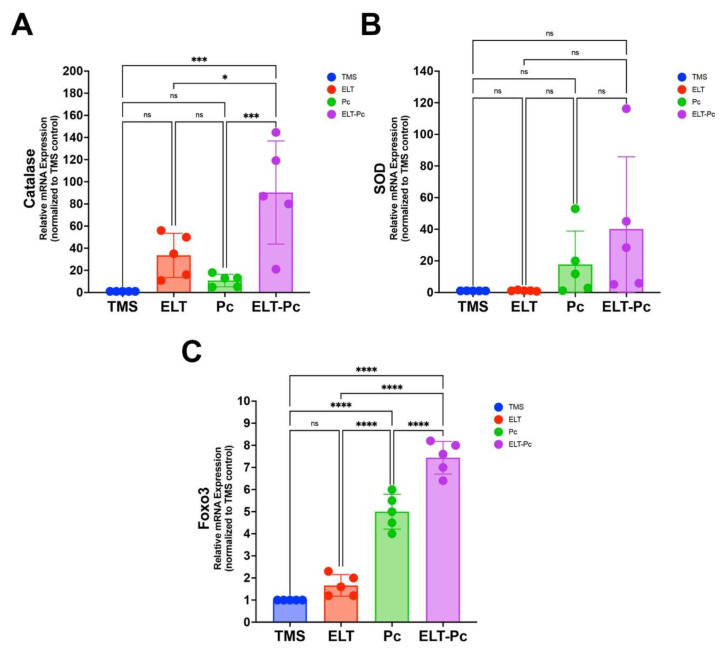
Evaluation of redox-response markers in elastase COPD-induced animals colonized with *Pneumocystis*. Catalase (**A**), Superoxide dismutase (SOD) (**B**), and Foxo3 (**C**) mRNA levels were measured by qPCR. Relative mRNA expression of each gene was determined using the 2^−DDCt^ method. Data were expressed as mean ± SD and analyzed by ANOVA. ns = non-significant; * = *p* < 0.05; *** = *p* < 0.001; **** = *p* < 0.0001.

Given the marked increment in inflammation around the airways, summed to the increment of the mRNA levels of inflammation markers and the pronounced airway remodeling observed across experimental groups, including epithelial thickening, mucus metaplasia, and increased peribronchial collagen deposition, we next examined whether these structural alterations were associated with activation of redox-responsive pathways in the lungs of these animals. To this end, the mRNA levels of key antioxidant genes were evaluated by qPCR (Figure 7). Animals exposed to *Pneumocystis* and elastase showed a marked and significant upregulation of catalase expression compared with the other groups (Figure 7A). In contrast, superoxide dismutase (SOD) mRNA levels did not differ significantly (Figure 7B). Evaluations of Foxo3 transcription factor mRNA levels revealed a different response pattern, with colonized animals showing a robust upregulation compared with controls, which was further amplified in the ELT-Pc group (Figure 7C). Collectively, these results indicate that *Pneumocystis* colonization may modulate redox-responsive signaling, thereby triggering an antioxidant response.

### 3.8. Pneumocystis Colonization of the Airways of Elastase-Induced COPD Animals Is Associated with Nrf2 Pathway Activation

**Figure 8:**
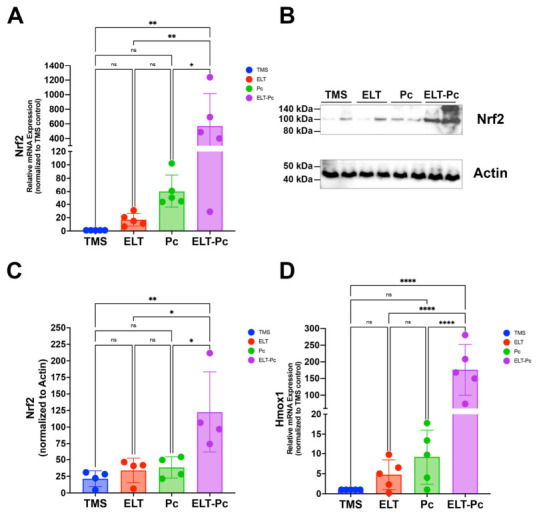
Evaluation of Nrf2 pathway status in the elastase-COPD-induced *Pneumocystis* colonized animal model. (**A**) Nrf2 mRNA levels were determined by qPCR, and its relative expression was determined using the 2^−DDCt^ method. (**B**) Representative Western blot to identify the protein levels of Nrf2 in protein extracts from the lungs of animals of all the experimental groups. Extracts from two animals per group are presented. Actin protein levels were evaluated as a loading control. (**C**) Quantification of Nrf2 Western blot, including the replicates shown in Figure S1 (n = 4). (**D**) mRNA levels of the downstream gene Hmox1 were determined by qPCR as described previously. Data from qPCR and Western blot experiments were expressed as mean ± SD and analyzed by ANOVA. ns = non-significant; * = *p* < 0.05; ** = *p* < 0.01; **** = *p* < 0.0001.

Given the enhanced antioxidant response observed in elastase-treated and *Pneumocystis*-colonized animals, we next examined whether the Nrf2 signaling pathway was activated under these conditions. To this end, Nrf2 mRNA levels were evaluated. These evaluations showed an increase in the Pc group compared with the control animals, but without significance (Figure 8A). In contrast, ELT-Pc animals showed a marked increase in Nrf2 mRNA levels, which was significantly higher compared with the TMS control group and single-hit groups. These same changes were observed when Nrf2 protein levels were analyzed by Western blot. A higher increase in protein levels was observed in the ELT-Pc group (Figure 8B,C and Figure S1). Nrf2 pathway direct activation was evaluated by measuring the downstream antioxidant target gene Hmox1 mRNA levels. The highest Hmox1 mRNA levels were observed in the COPD animals colonized with *Pneumocystis* compared with the other groups (Figure 8D). Together, these findings indicate that combined elastase-induced COPD and *Pneumocystis* colonization are associated with the activation of the Nrf2 antioxidant pathway.

### 3.9. Remodeling and Antioxidant Variables Are Associated with Pneumocystis Colonization in the Airways of Elastase-Induced COPD Animals: An Exploratory Approach

**Figure 9:**
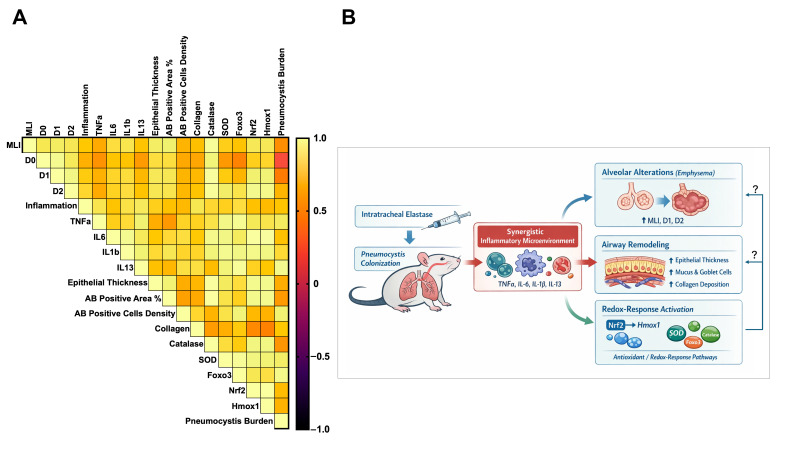
Correlation analysis integrating airway remodeling, inflammation, antioxidant response, and *Pneumocystis* burden in the context of COPD. (**A**) Heatmap representation of Pearson correlation coefficients among morphometric parameters of emphysematous injuries (MLI, D_0_, D_1_, and D_2_), inflammation markers (TNFα, IL6, IL8, IL1β, IL13), airway remodeling indices (epithelial thickness, AB-positive area, AB-positive cells density, and collagen deposition), redox-related gene expression (catalase, SOD, Foxo3, Nrf2, and Hmox1), and *Pneumocystis* burden. Correlation coefficients (r) were calculated using mean values for each variable and experimental group and are color-coded according to their magnitude and direction. Light colors represent positive correlation, and dark colors represent negative correlation. (**B**) Graphical abstract indicating the model of a putative mechanism induced by the colonization of *Pneumocystis* in COPD animals, generating a chronic inflammatory microenvironment in the airways, leading to the increment in alveolar damage, airway remodeling, and redox imbalance. It is not clear what mechanisms the redox imbalance may induce alveolar alterations and airway remodeling (question marks). Further investigations are needed to evaluate the causal relationships among these factors.

To further explore the relationships among structural lung remodeling, inflammation, redox response, and *Pneumocystis* colonization, a Pearson correlation analysis was performed, including all variables evaluated in this work (Figure 9A). As expected, strong positive correlations were observed among classical emphysema parameters, with MLI closely correlating with D_0_, D_1_, and D_2_ (r = 0.86–0.87), confirming their concordance in capturing alveolar airspace enlargement. These emphysematous injury parameters were also strongly associated with airway thickness and AB-positive epithelial area (r = 0.83–0.92), supporting the notion that parenchymal destruction and airway remodeling evolve in parallel in this model. Importantly, inclusion of *Pneumocystis* burden revealed moderate to strong positive correlations with multiple variables of lung remodeling. A moderate correlation between fungal burden and emphysematous damage parameters was identified: MLI (r = 0.50), D_1_ (r = 0.42), D_2_ (r = 0.66), and a very low correlation with the D_0_ index (r = 0.17). These results suggest a moderate association between fungal load and both alveolar alterations. Epithelial thickness and AB-positive epithelial area showed moderate to high correlations with *Pneumocystis* burden (r = 0.55–0.62). Interestingly, the strongest correlations involving *Pneumocystis* load were observed with parameters reflecting mucus-related remodeling and tissue reorganization, including AB-positive cells density (r = 0.92) and peribronchial collagen deposition (r = 0.74), indicating that higher *Pneumocystis* burden may be associated with enhanced airway remodeling and collagen accumulation. Additionally, *Pneumocystis* load exhibited robust positive correlation with antioxidant genes, including SOD (r = 0.90), Foxo3 (r = 0.96), Nrf2 (r = 0.69), and Hmox1 (r = 0.65). These associations mimic the strong correlations observed between remodeling parameters and antioxidant genes, particularly those related to the Nrf2 pathways (r = 0.77–0.96), indicating an association between antioxidant signaling and lung damage. In addition, remodeling parameters showed moderate to high correlations with antioxidant genes (r = 0.44–1.00).

In addition, the inclusion of inflammation-related variables further strengthens the integrative interpretation of these findings. Inflammation scores showed moderate to strong positive correlations with structural remodeling parameters, including MLI (r = 0.737), D1 (r = 0.750), and D2 (r = 0.796), as well as with epithelial thickness (r = 0.768) and collagen deposition (r = 0.860), supporting a close association between inflammatory processes and both parenchymal and airway remodeling. Similarly, inflammation correlated with antioxidant-related genes, including Catalase (r = 0.799), SOD (r = 0.770), Foxo3 (r = 0.825), Nrf2 (r = 0.707), and Hmox1 (r = 0.683), suggesting a link between inflammatory signaling and activation of redox-responsive pathways. Pro-inflammatory cytokines showed consistent correlations with both remodeling and redox variables. TNFα showed strong correlations with inflammatory score (r = 0.852) and IL13 (r = 0.914), as well as with antioxidant genes such as SOD (r = 0.948), Foxo3 (r = 0.969), Nrf2 (r = 0.872), and Hmox1 (r = 0.848). IL6 displayed particularly strong correlations with redox-response genes, including Nrf2 (r = 0.995) and Hmox1 (r = 0.991), and was also associated with emphysema-related parameters such as MLI (r = 0.826) and D1 (r = 0.790). Likewise, IL1β showed strong associations with both antioxidant genes (SOD: r = 0.965; Nrf2: r = 0.956) and remodeling markers (MLI: r = 0.852; epithelial thickness: r = 0.808). Notably, IL13, a cytokine closely associated with mucus production and airway remodeling, showed strong correlations with inflammation (r = 0.877), TNFα (r = 0.914), and IL1β (r = 0.916), as well as with mucus-related parameters such as AB-positive cell density (r = 0.833) and collagen deposition (r = 0.833). Importantly, IL13 also exhibited strong associations with antioxidant-related genes, including SOD (r = 0.928) and Foxo3 (r = 0.982), suggesting a link between type 2–associated inflammatory responses and activation of redox-responsive pathways. Importantly, *Pneumocystis* burden showed strong positive correlations with multiple inflammatory markers, including TNFα (r = 0.881), IL1β (r = 0.807), IL13 (r = 0.964), and global inflammation score (r = 0.727). In addition, *Pneumocystis* burden correlated with key remodeling parameters such as AB-positive cell density (r = 0.917) and collagen deposition (r = 0.738), as well as with antioxidant genes including SOD (r = 0.896), Foxo3 (r = 0.956), Nrf2 (r = 0.689), and Hmox1 (r = 0.649). These findings support a model in which fungal colonization is closely associated with an enhanced inflammatory microenvironment that may contribute to both structural remodeling and activation of redox-responsive pathways.

Collectively, this integrative, exploratory, correlational analysis shows an association among *Pneumocystis* colonization, lung structural remodeling, and activation of an antioxidant response pathway, consistent with the concept that fungal colonization is closely associated with the exacerbation of epithelial, parenchymal, and molecular alterations in the context of COPD. In this framework, inflammation emerges as a potential upstream mechanism linking fungal colonization with both tissue remodeling and redox imbalance, suggesting that *Pneumocystis*-associated inflammatory response may play a central role in modulating host redox signaling.

## 4. Discussion

In this study, we demonstrate that *Pneumocystis* exposure markedly exacerbates lung structural damage, airway remodeling, and redox-responsive signaling in a rat elastase-induced COPD model. While elastase administration alone recapitulated key features of emphysema and airway remodeling, the concomitant presence of *Pneumocystis* consistently amplified these pathological alterations, supporting the concept that fungal colonization acts as a disease modifier rather than a passive spectator in chronically injured lungs [65]. Interestingly, the presented results replicate some findings from this same COPD-induced elastase model but focus primarily on its characterization and features, such as inflammation and mucus hypersecretion [58,59]. This work adds new analysis and a new perspective on the role of *Pneumocystis* as an exacerbating factor in COPD.

One of the findings of this work is the worsening of emphysematous changes observed in animals exposed to both elastase and *Pneumocystis*. Classical morphometric indices, including MLI, D_0_, D_1_, and D_2_ parameters, revealed that combined injury led not only to increased average airspace enlargement but also to greater heterogeneity and severity of alveolar destruction. The use of “D” parameters strengthens these observations by capturing the distributional aspects of airspace enlargement, which are increasingly recognized as more sensitive indicators of emphysema progression than MLI alone [62]. The correlations observed among these indices further validate their concurrent assessment of parenchymal remodeling in this model. In addition, our data reveal a parallel, coordinated remodeling of the airways. Animals exposed to both elastase and *Pneumocystis* exhibited pronounced epithelial thickening, increased AB-positive epithelial area, and higher densities of mucus-producing cells. Although these findings are consistent with mucus cell remodeling and mucus metaplasia commonly observed in COPD [66], the magnitude of these changes in the combined injury groups suggests that *Pneumocystis* colonization intensifies epithelial responses beyond those induced by elastase alone. Importantly, although our data are compatible with goblet cell metaplasia, it is necessary to examine other mucus-secreting cell types, such as club cells. However, it has been reported that *Pneumocystis* may alter the phenotype of club cells toward a mucus-secreting phenotype in distal airways [67], and we previously documented the upregulation of mucin genes associated with mucus hypersecretion [59], supporting the metaplasia phenomenon.

A novel aspect of this study is the demonstration that *Pneumocystis* colonization correlates with multiple structural remodeling parameters. The inclusion of fungal burden in the correlation analysis revealed a robust association with mucus-related variables and collagen deposition, suggesting that higher levels of *Pneumocystis* are linked to enhanced airway remodeling and extracellular matrix reorganization. However, a limitation of this study is the absence of experiments demonstrating an increase in the fungal burden and its correlation with increased airway damage, such as dose- or time-dependent experiments, as previously reported [49]. This point may relate to the role of some immune cells relevant in adaptive response, which have been shown to control the burden of *Pneumocystis* in other models [36]. We previously showed that T- and B–cell levels did not change with increased *Pneumocystis* burden in an immunocompetent rat model [49]. Despite this point, these observations are consistent with emerging evidence that *Pneumocystis* prevalence, even in the absence of pneumonia, can be associated with chronic inflammatory and remodeling responses observed in chronic respiratory disease and animal models [48,49,58,59,68,69]. In this context, our data extend previous observations by positioning *Pneumocystis* as a potential amplifier of COPD-associated remodeling processes. The accumulation of peribronchial collagen, as revealed by Picrosirius red staining, further supports this concept. While elastase or *Pneumocystis* alone induced moderate increases in collagen deposition, the combined exposure resulted in the most pronounced fibrotic response. This finding is consistent with previous observations in a *Pneumocystis* primary infection model and in the same COPD elastase-induced model [49,58]. This result suggests that fungal-associated inflammatory or oxidative signals may promote fibroblast activation or extracellular matrix reorganization in an already susceptible COPD-like environment. Previously, we identified that an increase in collagen deposition around the airways may be triggered by activation of the profibrotic TGFβ1 pathway [58]. To strongly support this association between the fungus and extracellular matrix reorganization, it is necessary to incorporate specific remodeling markers, such as α-smooth muscle actin (α-SMA) and collagen I, to evaluate the fibrotic phenotype induced by *Pneumocystis* in this COPD-induced model.

Redox signaling emerges as a putative mechanism linking *Pneumocystis* exposure and lung remodeling in this animal model. The combined elastase-*Pneumocystis* group exhibited marked upregulation of antioxidant and redox-responsive genes, including catalase, Foxo3, Nrf2, and Hmox1. These responses were not only more pronounced than in single-injury groups but also strongly correlated with both structural remodeling indices and *Pneumocystis* burden. Activation of the Nrf2-Hmox1 axis is generally interpreted as a protective response to oxidative stress [70]; however, persistent or excessive activation may reflect sustained redox imbalance and ongoing tissue injury. In this regard, our findings suggest that *Pneumocystis* colonization contributes to a chronic oxidative microenvironment that may perpetuate epithelial dysfunction and remodeling, thereby modulating antioxidant pathways such as Nrf2. This point is supported by limited evidence that *Pneumocystis* may survive under oxidative conditions in a mouse cigarette smoke model [71]. In addition, genomic analysis indicates that *Pneumocystis* lacks a robust oxidative stress response pathway, creating a scientific gap [72]. Further evaluations of this fungus’s adaptive responses to oxidative stress are needed.

The integrative correlation analysis provides a systems-level view of how *Pneumocystis* burden, structural damage, inflammation, airway remodeling, extracellular matrix deposition, and redox responses are interconnected. While these correlations do not necessarily imply causality and are best viewed as exploratory, they support a model in which fungal colonization is closely associated with coordinated pathological processes across multiple lung compartments. This aligns with the concept that *Pneumocystis* may act as a new exacerbation agent and complements previous research on viruses, bacteria, and the fungus *Aspergillus* [73,74,75].

Some limitations of this study should be commented on. First, correlation analyses were performed using group-level mean values, which may hide individual variability and preclude causal inference. Second, although picrosirius red staining demonstrates increased collagen accumulation, additional molecular and cellular markers are required to fully define the fibrotic phenotype. Third, while redox-response markers were evaluated at the transcriptional and protein levels, direct measurement of ROS production or antioxidant enzyme activity was not performed. In addition, redox response enzymes were measured only at the mRNA level; it is necessary to determine their activity in this model to validate the presented observations. Moreover, a specific model related to the study of oxidative stress, that is, the cigarette smoke-induced COPD model, must be evaluated as a future direction of this work. Finally, extrapolation of these findings to human COPD patients must be done cautiously, as the dynamics of *Pneumocystis* colonization and host responses may differ between experimental models and patients. Moreover, in this context, functional analysis in animal models and patients must be performed to identify the impact of *Pneumocystis* colonization in COPD. However, we are confident that the current results, despite their limitations, are consistent with published evidence from our group and others.

In addition, inflammation is a key factor in airway remodeling in COPD and may be an important mechanism underlying these findings [23]. In addition, inflammation is a key factor in exacerbations, as documented, including the increment of inflammatory cytokines such as IL6 and IL8 in COPD patients. Moreover, the rapid increase in inflammation in these patients is principally due to bacterial pathogens (*H. influenzae*, *S. pneumoniae*, or *P. aeruginosa*) or viral pathogens (primarily Rhinovirus and influenza virus), which share acute airway inflammation. The inflammation parameters evaluated in this work showed a marked increase in COPD animals colonized with *Pneumocystis*, and, by correlation analyses, this variable is likely a key factor in the mechanism linking lung injury to the chronic colonization by the fungus. We previously documented a synergistic increase in inflammation markers, including classical markers such as NF-kβ related and IL1β signaling-related genes [58,59]. In addition, the Th2 immune response, including IL-13, was previously described as an important mechanism that induces mucus hypersecretion during *Pneumocystis* primary infection [48,49]. These elements are associated with mucus-related changes and extracellular matrix deposition, thereby promoting airway remodeling. Moreover, inflammation signaling is linked to the Nrf2 pathway, thereby increasing anti-inflammatory responses and crosstalk with the NF-κB pathway [76]. In this context, it is probable that *Pneumocystis* colonization may contribute to a proinflammatory microenvironment that secondarily amplifies both structural remodeling and redox-responsive signaling. While this study was not designed to directly evaluate inflammatory mechanisms, integrating inflammatory profiling with remodeling and redox analysis will be an important focus of future studies. Moreover, evaluation of a direct causal mechanism involving the Nrf2 pathway must include experiments using antioxidants or Nrf2 inhibitors. Taken together, we show that inflammation links remodeling and redox-responsive signaling in COPD using an exploratory approach, without a direct causal relationship. This evidence may be explained by lung damage induced by the exacerbation rather than by a redox imbalance, but further studies are needed to clarify this observation.

Finally, our findings show that enhanced airway remodeling and emphysematous injury, together with an antioxidant response, may reflect a putative compensatory response to the chronic lung-inflammation microenvironment induced by *Pneumocystis*. In this case, because COPD is induced by elastase rather than cigarette smoke, endogenous factors that increase the antioxidant response are more likely to be involved. The upregulation of antioxidant-associated genes may reflect an adaptive attempt to restore redox homeostasis rather than effective prevention of structural damage. In this context, *Pneumocystis* colonization may further intensify this process by amplifying epithelial and immune activation, increasing cellular stress burden, and thereby reinforcing both remodeling and redox-responsive signaling. Thus, activation of antioxidant pathways and progressive lung injury should be viewed as parallel and interconnected outcomes of chronic stress rather than mutually exclusive processes. However, these results support a model in which fungal colonization may contribute to a redox imbalance that may amplify the typical pathological features of COPD (Figure 9B). Although additional studies are required to establish a putative mechanism, these exploratory findings still support the role of *Pneumocystis* as an amplifying factor of COPD-like lung damage, with potential implications for understanding disease progression and identifying novel therapeutic targets.

## Figures and Tables

**Table 1:** Primers used in this work.

Gene		Sequence (5′–3′)	Size (bp)
*Dhfr*	Forward	GTTGCACTTACAACTTCTTATGG	223
	Reverse	TAGATCCAGAGATTCATTTCGAG	
*Actin*	Forward	CTTGCAGCTCCTCCGTCGCC	228
	Reverse	CTTGCTCTGGGCCTCGTCGC	
*Catalase*	Forward	AGAGGCAGGAAGACTTGCAC	178
	Reverse	GTCCTTGTGAGGCCAAACCT	
*SOD*	Forward	CGGGGGCCATATCAATCACA	162
	Reverse	CCTGAACCTTGGACTCCCAC	
*Foxo3*	Forward	GCTCCCTGCGAGTGTCTATAA	187
	Reverse	GAGGGCGTACGATTCCGC	
*Hmox1*	Forward	TCGACAACCCCACCAAGTTC	231
	Reverse	AGGTAGTATCTTGAACCAGGCT	
*Nrf2*	Forward	TGCTCCGACTAGCCATTGAC	140
	Reverse	ATCCATGTCCTGCTGGGACT	
*TNFα*	Forward	CAGCCGATTTGCCACTTATA	71
	Reverse	TCCTTAGGGCAAGGGCTCTT	
*IL6*	Forward	CCCAACTTCCAATGCTCTCCTAATG	141
	Reverse	GCACACTAGGTTTGCCGAGTAGACC	
*IL1β*	Forward	AGGCTTCCTTGTGCAAGTGT	202
	Reverse	TGTCGAGATGCTGCTGTGAG	
*IL13*	Forward	GTGGTCTTGCCACCCCAGGG	153
	Reverse	CGCCAGCTGTCAGGTCCACG	

## Data Availability

Any data presented in this study may be available on request from the corresponding author.

## Supplementary Materials

The following supporting information can be downloaded at: https://www.mdpi.com/article/10.3390/antiox15050526/s1, Table S1: Correlation of Multiples Variable; Figure S1: Original Western blot experiments.

## Abbreviations

The following abbreviations are used in this manuscript:

COPD	Chronic obstructive pulmonary disease
MLI	Mean linear intercept
SOD	Superoxide dismutase
Nrf2	Nuclear factor erythroid 2-related factor 2
Hmox1	Heme oxygenase 1
ROS	Reactive oxygen species
ELT	Elastase
TMS	Trimethoprim–sulfamethoxazole
*dhfr*	Dihydrofolate reductase
H/E	Hematoxylin/eosin
AB	Alcian blue
Pc	*Pneumocystis carinii*
