# Supplementary material for: Pneumocystis Colonization Is Associated with Enhanced Pulmonary Remodeling and Activation of Redox-Responsive Pathways in a COPD Experimental Model
Source: Antioxidants (Basel). 2026 Apr 22;15(5):526. doi: 10.3390/antiox15050526 (PMC13203691; doi:10.3390/antiox15050526)
Supplement: Supplementary file 1 [file antioxidants-15-00526-s001.zip › Figure S1.pdf]

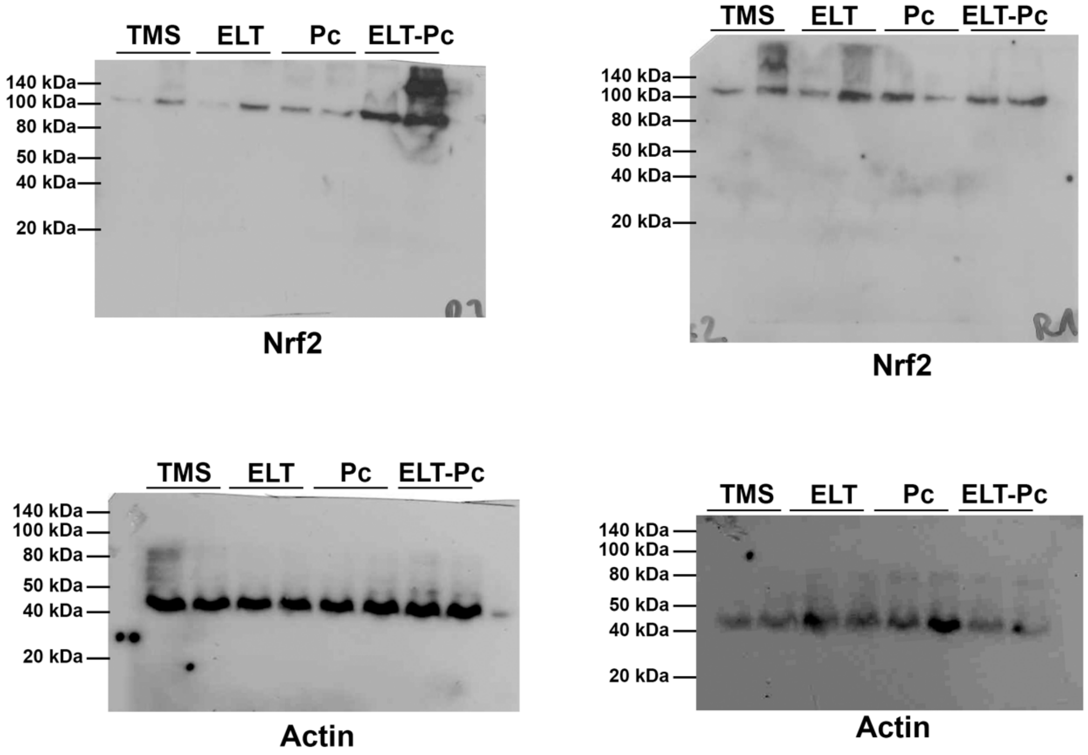

Figure S1. Original Western blot experiments. Protein levels of transcription factor Nrf2 were measured. Two representative protein extracts are indicated for each experimental group, including two replicates. Actin was used as an internal control. Improved images of these experiments, along with replicate quantification, are shown in Figure 7B and 7C.
